# Formulation of Ascorbic Acid and Betaine-based Therapeutic Deep Eutectic System for Enhanced Transdermal Delivery of Ascorbic Acid

**DOI:** 10.3390/pharmaceutics16050687

**Published:** 2024-05-20

**Authors:** Ji-Eun Song, Seung-Hyun Jun, Joo-Yeon Ryoo, Nae-Gyu Kang

**Affiliations:** R&D Center, LG Household and Health Care (LG H&H), 70, Magokjungang 10-ro, Gangseo-gu, Seoul 07795, Republic of Korea; sos6934@lghnh.com (J.-E.S.); ryoojy93@lghnh.com (J.-Y.R.)

**Keywords:** ascorbic acid, betaine, therapeutic deep eutectic solvent, transdermal delivery, solubility, gluconolactone

## Abstract

L-ascorbic acid (AA), a potent antioxidant, is commonly used topically in the pharmaceutical and cosmetic fields. However, the incorporation of AA into topical formulations is difficult because of its highly unstable nature and relatively poor skin permeability. In this study, we propose an alternative strategy for improving the solubility and topical delivery of AA through its conversion to a therapeutic deep eutectic system (THEDES). AA and betaine (Bet)-based THEDESs were prepared at certain molar ratios and characterized using polarized optical microscopy, Fourier transform infrared spectroscopy, and differential scanning calorimetry. Solubility tests showed that AA in the form of THEDES was readily soluble in various polyols (glycerin, 1,3-butylene glycol, dipropylene glycol, and 1,3-propanediol) at a high concentration (approximately 40%). Furthermore, compared to AA alone or the physical mixture of AA and Bet, AA-based THEDES significantly enhanced AA delivery through porcine skin. In an in vivo human study, THEDES-containing serum reduced the markers of aging and induced an even skin tone. These findings indicate the utility of AA and Bet-based THEDES as novel transdermal delivery systems for AA. Furthermore, our approach also showed good extension to developing gluconolactone, a well-known natural antioxidant, and Bet-based THEDES, showing potential application in transdermal delivery systems.

## 1. Introduction

Deep eutectic solvents (DESs), introduced by Abbott et al. in 2003, are formed by mixing two or more hydrogen bond donor and acceptor components through the hydrogen bonding interactions and possess significant melting point depressions relative to the individual components [1,2,3,4]. They have recently been considered versatile alternatives to ionic liquids (ILs) owing to several significant advantages, such as simple preparation, nontoxicity, low cost, and non-necessity for purification, and IL-like physicochemical properties (such as low melting point, low vapor pressure and volatility, good thermal stability, and tunable polarity) [5]. Furthermore, DESs are composed of plant-based metabolites, such as sugars, alcohols, amino acids, and organic acids, and were studied and indicated as natural DESs (NADESs) by Choi et al. in 2011 [6]. Industrial applications of NADESs are highly promising and include extraction [7,8], nanomaterial synthesis [9,10], enzymatic reactions [11], cosmetic formulations [12,13], and pharmaceutical formulations [14,15]. Regarding pharmaceutical formulations, DESs composed of at least one active pharmaceutical ingredient (API) in their structure are called therapeutic DESs (THEDESs), a recently emerging type of DES. Notably, several studies have reported significant improvements in skin permeation, intestinal absorption, solubility, and controlled release of APIs in the form of THEDES [16]. Stott et al. were the first to report a THEDES system composed of ibuprofen as a drug and different terpenes as permeation enhancers; the formed THEDESs enhanced transdermal drug delivery [17]. Similarly, THEDES containing aspirin–-choline chloride was reported to be miscible with water, although aspirin has poor solubility in water [16]. Furthermore, the development of multifunctional drug delivery systems based on DESs formed by two different APIs, such as lidocaine–ibuprofen [18] and paeonol–osthole [19] has been investigated for the co-delivery of dual drugs with enhanced solubility and transdermal delivery. However, only a few studies have investigated THEDESs formed by the two APIs.

L-ascorbic acid (AA; vitamin C), a well-known water-soluble vitamin, has extremely low passive permeability owing to its intrinsic hydrophilicity (log K_ow_ = −2.13) and the characteristics of the stratum corneum (SC) of the skin [20,21]. Various strategies to facilitate skin penetration of AA have been studied, such as encapsulation of AA in soluble microneedles [22] or hydrogels [23] or the use of iontophoresis techniques [24]. AA is widely recognized as a compound with low stability that is prone to degradation by air, moisture, light, heat, and metal ions. Its degradation is typically characterized by yellow discoloration and can be accelerated depending on the formulation, packaging, and storage conditions [25,26,27,28]. Various stabilization strategies have been attempted to address the instability of AA, such as liposomal encapsulation [29,30], anhydrous suspensions [31], and the use of vitamin C derivatives [32,33]. Despite its low skin permeability and instability, AA remains widely used in dermatology and cosmetic formulations as a skin-whitening and anti-aging agent [34,35], owing to its excellent antioxidant properties, ability to inhibit melanin synthesis [36,37], and promotion of collagen formation [38,39].

**Scheme 1:**
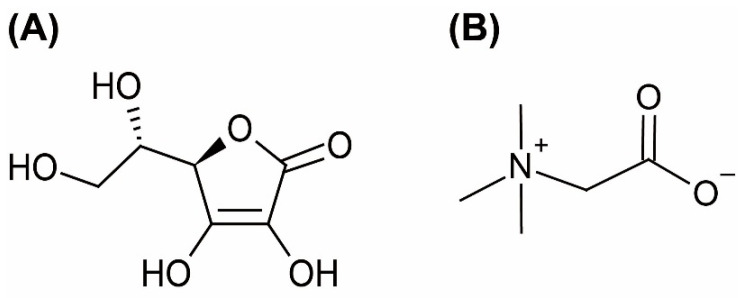
Chemical structure of (**A**) L-ascorbic acid and (**B**) betaine.

Topical applications of AA are limited by its low skin permeability and poor stability in aqueous conditions. Furthermore, the low solubility of AA in non-aqueous polyol solvents makes it difficult to minimize the amount of water required to stabilize AA. To address these limitations, we introduced another hydrophilic API, betaine (Bet; log K_ow_ = −4.9), for THEDES formation with AA. The chemical structures of the two APIs are shown in Scheme 1. Bet, a metabolite of choline, is widely present in nature, including sugar beets and marine life [40,41,42]. It has moisture-retentive, anti-oxidative, and anti-inflammatory properties [43]; therefore, it is used for skin moisturization [44], reduction of oral mucosa irritation [45], and wound healing [43]. In the present study, a new AA and Bet-based THEDES was constructed to enhance the properties of AA, particularly its solubility in water and non-aqueous polyol solvents and its skin permeability.

## 2. Materials and Methods

### 2.1. Materials

L-Ascorbic acid (99.5%) was purchased from Daejung Chemical Co. (Seoul, Republic of Korea). Gluconolactone was purchased from Roquette Frères (Lestrem, France). Betaine was purchased from Asahi Kasei Chemicals (Tokyo, Japan). Glycerin was obtained from LG H&H (Ulsan, Republic of Korea). 1,3-Butylene glycol was purchased from Oxea Chemicals (Dallas, TX, USA). Potassium hydroxide was purchased from Youngjin Co. (Bucheon, Republic of Korea). Phosphate-buffered saline (PBS) was purchased from GIBCO (Grand Island, NY, USA). Dipropylene glycol was purchased from SKC (Suwon, Republic of Korea), and 1,3-propanediol was purchased from DuPont Tate & Lyle Bio (Loudon, TN, USA). Phosphoric acid was purchased from Junsei (Tokyo, Japan), and potassium dihydrogen phosphate was purchased from Sigma Aldrich (St. Louis, MO, USA).

### 2.2. Preparation of AA:Bet:H_2_O THEDES

THEDES (TheraC) was prepared by heating and stirring. The three components (AA, Bet, and water) were mixed in different molar ratios, as presented in Table 1. The mixtures were heated to 80 °C under constant stirring at 500 rpm until a clear liquid was formed (10 min).

### 2.3. Characterization of AA:Bet:H_2_O THEDES

#### 2.3.1. Polarized Optical Microscopy Analysis

Small droplets of the prepared DESs were deposited on glass slides for macroscopic optical characterization. Microscopic images under polarized light were acquired using a Leica DM1000 polarizing microscope (Leica, Wetzlar, Germany) equipped with a Leica DFC295 camera at room temperature (27 °C). Uniform black images confirmed the successful formation of a deep eutectic liquid without any crystal-like structures [46].

#### 2.3.2. Fourier Transform Infrared Spectroscopy Analysis

To analyze the prepared THEDES, Fourier transform-infrared (FT-IR) spectra were recorded at room temperature in the 4000–400 cm^−1^ range using a PerkinElmer Spectrum Two FT-IR spectrometer (Waltham, MA, USA).

#### 2.3.3. Differential Scanning Calorimetry Analysis

The THEDES and pure APIs (AA and Bet) were characterized using differential scanning calorimetry (DSC; DSC 4000, Perkin Elmer, Wellesley, MA, USA). The measurements were performed under a nitrogen atmosphere (at a flow rate of 50 mL/min), with samples of 5–10 mg packed in aluminum pans. The thermal characteristics of the samples were measured from −40 °C to 230 °C for the DES, from 30 °C to 230 °C for AA, and from 30 °C to 325 °C for Bet, at a heating rate of 5 °C/min, after equilibration time of 5 min.

#### 2.3.4. Solubility Measurement in Polyols

To determine the solubility of AA in polyols (glycerin, 1,3-propanediol, dipropylene glycol, and 1,3-butylene glycol), 75 g of the THEDES (AA:Bet:H_2_O at a molar ratio of 1:1:2; 40.1 g of AA, 26.7 g of Bet, and 8.2 g of water) was added to 25 g of the polyols, and the resultant samples were kept at room temperature under constant stirring for 4 h. Then, the samples were centrifuged (Centrifuge 5427R, Eppendorf, Germany) at 13,000 rpm for 30 min, and the supernatants were serially diluted using deionized water and were then filtered through 0.45 μm membrane filters. For comparison, the same mass of AA and water (molar ratio of 1:2; 40.1 g of AA and 8.2 g of water) was added to 25 g of the polyols, and the same procedure was performed. Then, 10 μL of the samples were subjected to high-performance liquid chromatography (HPLC). The HPLC system (Nexera Ultra-High-Performance Liquid Chromatography, Shimadzu, Japan) comprised a DGU-405 degassing unit, an LC-40D XR binary pump, and an SPD-M40 photodiode array detector. The HPLC column was the YMC-Triart C18 150 mm × 4.6 mm with an internal particle size diameter of 5 μm (YMC, Milford, MA, USA). The column temperature was set to 25 °C, and the injection volume was 10 μL. The mobile phase consisted of 20 mM of potassium dihydrogen phosphate–phosphoric acid (pH 2.5) with a flow rate of 1 mL/min. The ultraviolet absorbance of the AA was measured at 245 nm.

### 2.4. In Vitro Skin Permeation Studies

An in vitro permeation study was conducted using Franz diffusion cells. Porcine cadaver skin (2.5 cm × 2.5 cm × 1 mm) with approval from APURES Institutional Animal Care and Use Committee (IACUC, approval number: APURES-IACUC 220418-001) was obtained from APURES Co., Ltd. (Pyeongtaek, Republic of Korea) for an in vitro skin permeation study. Three groups of 30 wt% AA solutions were used; AA-alone, physical mixture, and THEDES groups. In the THEDES group, 5.07 g of AA:Bet:H_2_O (molar ratio of 2:1:6) THEDES was added to 4.93 g of glycerin and mixed for 30 min at room temperature. The physical mixture was prepared by sufficiently mixing the same composition as that of the THEDES-containing solution (3.00 g of AA, 1.00 g of Bet, 0.92 g of water, and 4.93 g of glycerin) for 30 min at room temperature. In the AA-alone group, 3.00 g of AA and 0.92 g of water were added to 5.93 g of glycerin, and the resultant solution was sufficiently mixed for 30 min at room temperature. All three solutions contained 0.15 g of potassium hydroxide to adjust the pH of the solutions. In all groups, a total of 100 mg of the solution containing 30.0 wt% AA was applied to porcine skin (*n* = 2), and the skin and receptor media were withdrawn after 5 h of application. To determine the amount of sample absorbed by the SC, the tape-stripping method was used to isolate the SC layer from the epidermis/dermis layer. The porcine skin was tape-stripped three times using a D-Squame pressure applicator and stripping tape (CuDerm, Dallas, TX, USA). Each tape was collected in an individual 1.5 mL Eppendorf tube, followed by the addition of 1 mL of PBS solution into the tube, which was maintained for 4 h to sufficiently extract the AA absorbed by the SC. The solutions were collected to determine the amount of AA absorbed by the SC. The SC-free porcine skin (epidermis/dermis layer) was homogenized in PBS solution (1 mL) using a Precellys 24 homogenizer (Bertin Technologies, Montigny, France) and centrifuged (Centrifuge 5427R, Eppendorf, Germany) at 13,000 rpm for 10 min. Each supernatant was collected and diluted 5 times with PBS to determine the amount of AA that permeated the epidermis/dermis layer. AA content was measured using a K-ASCO 11/05 Megazyme colorimetric kit (Megazyme International Ireland, Bray, Ireland) following the recommended procedure. Briefly, in the presence of an electron carrier at pH 3.5, L-ascorbic acid (L-ascorbate) in the sample reduced the tetrazolium salt to a formazan compound, as measured by the increase in absorbance at 578 nm. The absorbance was measured at 578 nm using a multifunctional microplate reader (Varioskan LUX, Thermo Scientific, Waltham, MA, USA).

### 2.5. In Vivo Efficacy Test

The in vivo efficacy test was performed on 10 healthy women aged 25–43 years (mean age: 32 years). This study was approved by the LG Household and Healthcare Institutional Review Board (IRB; LGHH-20211028-AA-03-01). All the participants provided IRB-approved informed consent before participation in the study. Clinical evaluation was performed before (day 0, baseline) and 14 and 28 days after daily use of THEDES-containing serum (23.0 wt% AA and 7.7 wt% Bet). Evaluation data on skin brightness, cheek elasticity, and image analysis of the nasolabial fold area were collected. Before measurement, the participants cleaned their faces and rested for 20 min at a constant temperature (22 °C) and humidity (50%). Cutometer^®^ MPA 580 (Courage + Khazaka electronic GmbH, Cologne, Germany) was used to measure the net elasticity of the skin, indicated as R5. The skin brightness (L* value) was measured using a CR-400 chroma meter (Konica Minolta, Osaka, Japan). The degree of wrinkling was measured with Antera 3D (Miravex, Dublin, Ireland) using the indentation index from images with a large filter (0–3 mm).

## 3. Results and Discussion

### 3.1. Polarized Optical Microscopy Analysis

**Figure 1:**
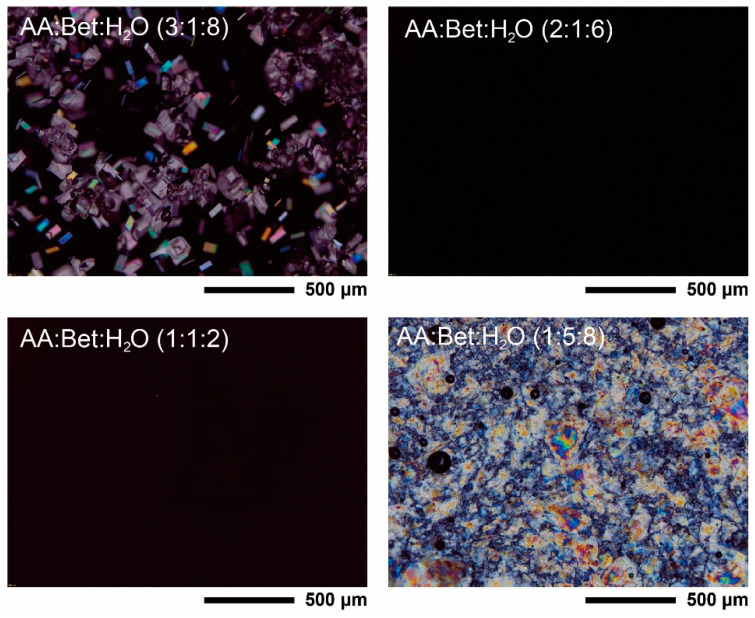
Polarized optical microscopy images of AA:Bet:H_2_O mixtures at different molar ratios after heating and stirring.

The THEDESs, prepared with AA, Bet, and a small amount of water at different molar ratios, as presented in Table 1, were macroscopically characterized using polarized optical microscopy (POM) to detect the presence of different phases, such as crystals. As shown in Figure 1, uniform black images were obtained at specific molar ratios of AA to Bet (2:1 and 1:1), confirming the formation of a homogeneous liquid without any crystal-like structures. Furthermore, the mixture of AA and Bet at molar ratios of 3:1 and 1:5 formed a viscous solid paste instead of a transparent liquid, and many crystal-like structures were observed in the POM analysis. However, since the mixture of AA and Bet at a molar ratio of 2:1 should additionally contain KOH (Table 1) and the amount of H_2_O is higher, the following studies were mainly conducted with the mixture of AA and Bet at a molar ratio of 1:1.

### 3.2. Fourier Transform Infrared Spectroscopy Analysis

**Figure 2:**
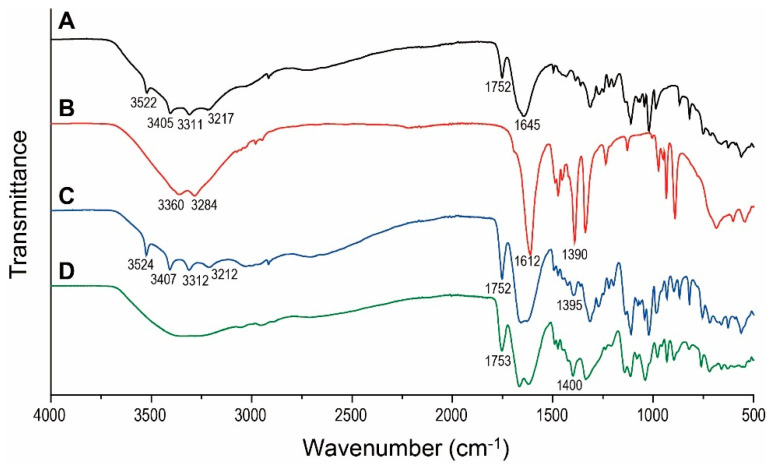
FT-IR spectra of (**A**) AA:H_2_O (1:2), (**B**) Bet:H_2_O (1:2), (**C**) the physical mixture, and (**D**) the THEDES (AA:Bet:H_2_O at a molar ratio of 1:1:2).

The formation of THEDES and the intermolecular interactions between the components were investigated using FT-IR spectroscopy. As shown in Figure 2, FT-IR spectra of AA, Bet, and their physical and eutectic mixtures containing equal molar amounts of water were measured in the wavenumber region of 500–4000 cm^−1^. Figure 2A shows the spectrum of the mixture of AA and water (AA:H_2_O at a molar ratio of 1:2), in which the characteristic bands of AA corresponding to the stretching vibrations of C=C and C=O were identified at 1645 cm^−1^ and 1752 cm^−1^, respectively [47]. There are four peaks for O-H vibrations of AA: 3217, 3311, 3405, and 3522 cm^−1^ [48]. Figure 2B shows the FT-IR spectrum of the mixture of Bet and water (Bet:H_2_O at a molar ratio of 1:2), which exhibits two clear peaks at 1612 cm^−1^ and 1390 cm^−1^ for COO^−^ and C-N stretching vibrations, respectively, confirming the characteristics of Bet [49,50].

For comparison with the eutectic mixture, a physical mixture with the same composition as the eutectic mixture (AA:Bet:H_2_O at a molar ratio of 1:1:2) was measured. The physical mixture was prepared by sufficiently mixing the same composition as that of the eutectic mixture at room temperature. The formation of THEDES has been reported to rely on hydrogen bonding [51,52]. In infrared spectroscopy, alterations in the bond length and the corresponding vibrations of hydrogen bonds may indicate the formation of hydrogen bonds [53]. The spectra of the physical and eutectic mixtures (Figure 2C,D) approximately overlapped those of AA:H_2_O and Bet:H_2_O; however, some spectral changes were observed in the THEDES compared with the physical mixture. The bands for hydroxyl groups in the spectra of AA:H_2_O and the physical mixture (Figure 2A,C) became broader and wider after eutectic formation in the range of 3200 cm^−1^ to 3550 cm^−1^ (Figure 2D). This spectral change indicates that many hydrogen bonds were formed between AA and Bet during the formation of THEDES. Furthermore, the C-H stretching vibrations observed at 3360 cm^−1^ and 3284 cm^−1^ in Figure 2B disappeared when THEDES was formed, suggesting the presence of hydrogen bonds. In addition, HPLC (Figure S1) and nuclear magnetic resonance spectrometry measurements (Figure S2) confirmed that AA and Bet are present in THEDES, which involves physical interactions rather than chemical reactions.

### 3.3. Differential Scanning Calorimetry and Solubility Studies

**Figure 3:**
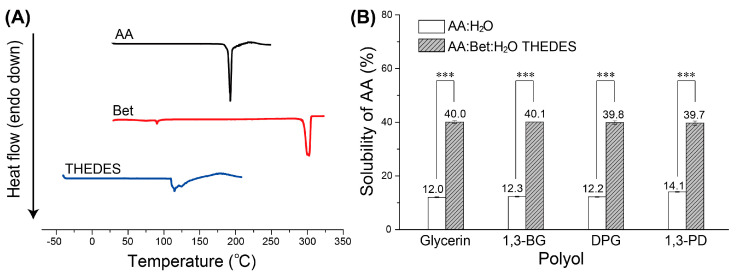
(**A**) DSC thermograms of AA, Bet, and the THEDES (AA:Bet:H_2_O at a molar ratio of 1:1:2). (**B**) The solubility (%) of AA in AA:H_2_O (molar ratio of 1:2) and the THEDES (AA:Bet:H_2_O at a molar ratio of 1:1:2) in glycerin, 1,3-butylene glycol (1,3-BG), dipropylene glycol (DPG), or 1,3-propanediol (1,3-PD). *** *p* < 0.001, a statistically significant difference between THEDES and AA:H_2_O.

The THEDES was characterized using DSC to determine the presence of thermal transitions, and AA and Bet were measured in powder form (Figure 3A). Pure AA and Bet showed endothermic peaks because of their melting points at 192.4 °C and 302.1 °C, respectively. Furthermore, the THEDES in the liquid phase did not present any peaks at the temperature range of −40 °C to 110 °C, and only a peak caused by water evaporation was observed at approximately 110 °C. This finding indicates that the THEDES lacked crystallinity and was stably maintained in a liquid state even at sub-zero temperatures (−40 °C).

Because AA is highly unstable in aqueous conditions, minimizing the amount of water and partially replacing the solvent with polyols to prepare a stable formulation are necessary. In addition, because high concentrations of AA formulations are effective for topical absorption [54], we investigated whether the solubility of AA in polyols could be improved through the formation of THEDES. The selection of four polyols (glycerin, 1,3-butylene glycol, dipropylene glycol, and 1,3-propanediol) as the solvents evaluated in this study was motivated by the importance of these solvents in the cosmetics and pharmaceutical industries and their frequent use as solvents or surfactants in topical formulations or pharmaceutical excipients [55]. In addition, THEDES is immiscible in certain oil solvents such as palmester oil and silicone oil; therefore, the polyols were evaluated as being miscible with water and many other polar solvents. Glycerin is primarily used as a humectant rather than a permeation enhancer, but it is believed to increase stratum corneum hydration, which may increase the fluidity of lipids and affect the permeation of active ingredients [56]. 1,3-Propanediol is a natural solvent, and its solvency properties enable effective skin delivery of certain active ingredients [57]. DPG and 1,3-BG are also solvents used in many topical formulations, primarily as humectants and preservative efficacy boosters.

As shown in Figure 3B, the solubility of AA in THEDES in various polyols was evaluated at room temperature (27 °C). Briefly, 75.0 g of THEDES (AA:Bet:H_2_O at a molar ratio of 1:1:2) was added to 25 g of polyols. The solubility of AA in the polyols was measured as a control. However, because THEDES contains a small amount of water and because the solubility of AA in water is high, the same mass of AA and water in THEDES was added to the polyols as a control sample (AA:H_2_O at a molar ratio of 1:2). We observed approximately a 2.8–3.3-fold increase in the solubility of AA in the form of THEDES (for example, 12.0% to 40.0% AA in glycerin) compared to AA:H_2_O. The solubility of AA in glycerin, 1,3-butylene glycol, dipropylene glycol, and 1,3-propanediol was 40.0%, 40.1%, 39.8%, and 39.7%, respectively. These results indicate that the solubility of AA in polyols was significantly enhanced when applied in the form of THEDES. Therefore, preparing a polyol-based high-concentration AA formulation with minimal water and increasing the stability of AA compared with that in a water-based formulation is possible. Furthermore, the formulation stability at low temperatures can be improved because the liquid phase is maintained even at low temperatures.

### 3.4. In Vitro Skin Permeation Studies

**Figure 4:**
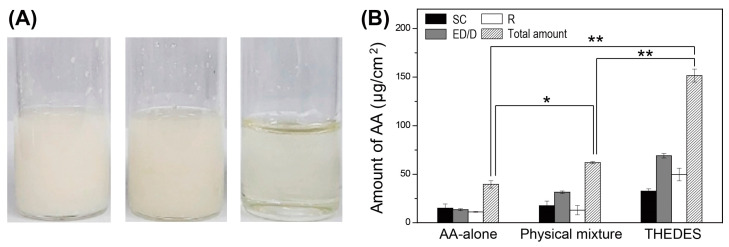
(**A**) Images of the three types of AA solutions used for in vitro skin permeation: AA-alone solution (left), physical mixture (middle), and THEDES (right). (**B**) Compartmental distribution of AA permeated into the stratum corneum (SC), epidermis, dermis (ED/D), and receptor (R) after 5 h of application in the three groups of AA solutions. Mean ± SD, *n* = 2. * *p* < 0.05, ** *p* < 0.01, a statistically significant difference between AA-alone, physical mixture, and THEDES groups.

An in vitro skin permeation study was conducted to investigate the pure skin permeability of the THEDES, which was quantitatively evaluated using the Franz diffusion cell technique. Porcine skin with thickness of 1 mm, composed of the stratum corneum, epidermis, and upper dermis, was used in this experiment. The porcine skin used is the skin from the back of micropig, which has morphological and functional characteristics similar to those of human skin, so it is a reliable animal model for evaluating transdermal absorption [58]. The receptor chamber was filled with 2.5 mL of PBS (pH 7.4). After mounting the skin on a Franz diffusion cell, THEDES (AA:Bet:H_2_O) in glycerin (THEDES group) was applied to the skin and placed in an incubator at 31 °C. A total of 100 mg of the solution containing 30.0 wt% AA was applied to 2.54 cm^2^ of the skin; therefore, the applied dose of AA was calculated as 11.8 mg/cm^2^. After 5 h of permeation, the applied solution was cleaned off, and the amount of AA that permeated the skin was measured. For comparison, a physical mixture of AA, Bet, and water in glycerin, with the same composition as that of the THEDES, was used. Furthermore, the AA single-component solution (30.0 wt%) without Bet (AA-alone group) was also used for comparison. Water could modify the solubility and skin permeability of AA; therefore, the same amount of water (9.2 wt%) as that in THEDES was added. As shown in Figure 4A, because of the low solubility of AA in glycerin (Figure 3B), the AA-alone and physical mixture groups were applied in suspensions, with white powders dispersed, unlike a transparent THEDES-containing solution.

We analyzed the relative distribution of AA in the porcine skin and receptor according to the three groups after 5 h of application (Figure 4B). The THEDES group showed an overall AA delivery of 151.6 ± 6.6 µg/cm^2^ in the skin and receptor, which was 3.8- and 2.4-fold higher than that of the AA-alone (39.6 ± 3.8 µg/cm^2^) and physical mixture (62.0 ± 1.1 µg/cm^2^) groups, respectively; Of the amount of AA applied (11.8 mg/cm^2^), 0.34%, 0.53%, and 1.28% of AA were absorbed into the skin and receptor in the AA-alone, physical mixture, and THEDES groups, respectively. In particular, in the AA-alone group, small amounts of AA were present in the epidermis/dermis (13.4 ± 1.0 µg/cm^2^) and receptors (11.1 ± 0.4 µg/cm^2^), whereas in the THEDES group, 69.2 ± 2.4 µg/cm^2^ and 49.8 ± 6.6 µg/cm^2^ of AA were present in the dermis and receptors, respectively. In other words, a large amount of ascorbic acid was delivered deep into the skin in the THEDES group, compared to the AA-alone group. The same amount of AA was applied; however, the difference in the amount on the skin might be due to the increased solubility of AA in polyols and a significant decrease in the melting point of AA by the THEDES formation. Generally, the primary basis of transdermal permeation enhancement using DES is to lower the melting point of the permeant [59]. According to the ideal solution theory [17], depressing the melting point of the permeant increases its solubility in skin lipids, suggesting that the enhanced skin permeability of AA in the form of THEDES is because of the increased solubility of THEDES in the stratum corneum. Because the stratum corneum is the major barrier to the transdermal delivery of AA [60], the increased permeability of THEDES into the stratum corneum significantly enhanced the amount of AA permeated below the stratum corneum in the THEDES group compared with the AA-alone group. The physical mixture group showed better AA delivery than the AA-alone group, possibly because the physical mixture contained a small amount of THEDES. Therefore, during the process of mixing the components at room temperature to prepare the physical mixture, the two APIs could react to form THEDES. However, the fact that the physical mixture was not entirely THEDES is also evident from the FT-IR spectrum (Figure 2). The permeation-enhancing effects of the THEDES were further evaluated at different concentrations (8, 15, 23, and 30% of AA). In addition, the skin permeability of AA increased in a concentration-dependent manner by approximately 30%; however, a higher degree of AA permeation was confirmed when compared to the aqueous solution-based commercial product (Figure S3). Overall, these results indicate that AA and Bet-based THEDES can effectively deliver large quantities of AA.

To further demonstrate the extendibility of our THEDES system, we developed another THEDES comprising Bet and gluconolactone (GCL). GCL, a polyhydroxy acid, is a natural antioxidant that has various properties, such as antioxidation/chelation, skin hydration, skin barrier strengthening, and gentle exfoliation [61]. The in vitro skin permeation of GCL was studied using pure GCL and DES formed by heating and mixing GCL, Bet, and water. The eutectic mixture showed significant enhancement of skin permeation, which was 1.4-fold higher than that of pure GCL (Figure S4A). Furthermore, compared with those of the control (untreated) and pure GCL, GCL in the DES form increased the daily average exfoliation rates by 31% and 19%, respectively (Figure S4B,C). This indicates that THEDES facilitates skin exfoliation by promoting the enhanced delivery of active ingredients into the skin. These results suggest that the system can also be applied to other APIs.

### 3.5. In Vivo Skin Changes after THEDES Serum Use

**Figure 5:**
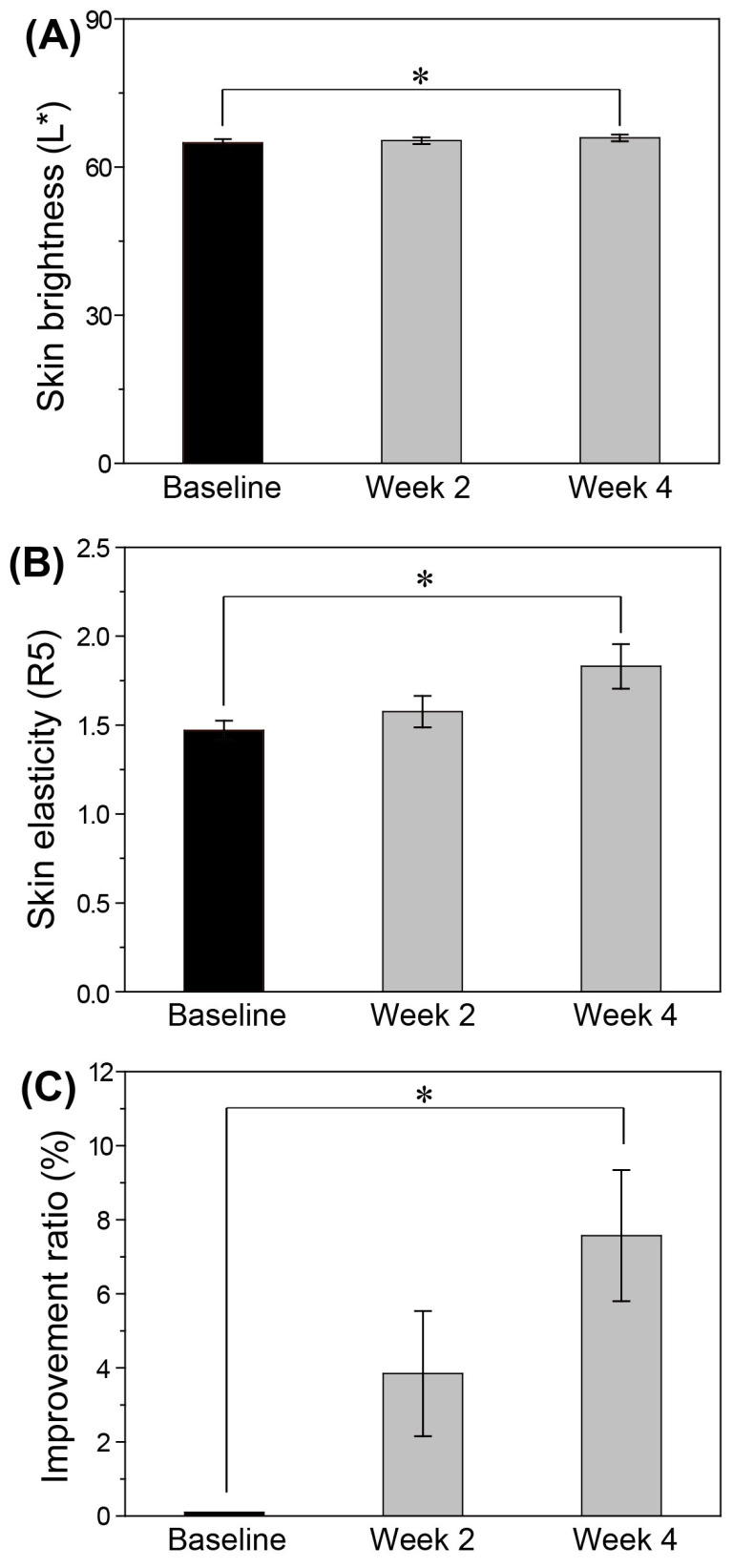
Comparison of skin parameters after daily serum use for 4 weeks. (**A**) Skin brightness, (**B**) Skin elasticity (R5), and (**C**) Improvement ratio of indentation index, which is used to measure the degree of wrinkling using Antera 3D^®^, were evaluated. Notes: The results are presented as Mean ± SE. * *p* < 0.05, statistically significant compared with baseline (day 0). R5, net elasticity of the skin.

The efficacy of the THEDES was confirmed by conducting an in vivo human study in 10 healthy females aged 25 to 43 years with various skin types (Table S1). The THEDES-containing serum, applied as a topical formulation, contains 23.0 wt% AA and 7.7 wt% Bet, and 1,3-propanediol as the primary solvent, and dipropylene glycol, glycerin, and water as co-solvents. The skin-brightening and anti-aging effects of the THEDES-containing serum were evaluated in female participants after applying the formulation to facial skin for 2 and 4 weeks. For skin brightening (Figure 5A), the THEDES-containing serum improved skin brightness after daily application during all test periods. The participants presented with brighter skin after 2 weeks of application (L* = 65.33, *p* = 0.20) and significantly brighter skin after 4 weeks of application (L* = 65.89, *p* < 0.05) compared to their skin brightness at baseline (L* = 64.88). Since THEDES enhanced the skin absorption of AA, it seems to be effective for improving skin brightness.

As shown in Figure 5B, the skin elasticity (R5) of the cheek area increased by 7.2% after 2 weeks (1.58 ± 0.09) and significantly increased by 26.1% (*p* < 0.05) after 4 weeks (1.83 ± 0.13) compared with day 0 (1.47 ± 0.06). Regarding the anti-aging effect, the wrinkle indentation index decreased in the nasolabial fold (by 3.8%) at 2 weeks compared with baseline. Furthermore, the serum significantly decreased wrinkling at 4 weeks (by 7.6%, *p* < 0.05) compared with baseline (Figure 5C). Evaluation of the changes in skin brightness and anti-aging effects suggested that THEDESs promote skin whitening, elasticity, and wrinkle improvement. However, further studies are needed to compare the efficacy of THEDES and AA alone on skin.

## 4. Conclusions

In this study, we designed a new THEDES based on two well-known APIs, AA and Bet, and water for topical application. THEDES formation dramatically increases the solubility of AA in polyol solvents, enabling the preparation of highly concentrated AA topical formulations with minimal water. Furthermore, the skin permeability of THEDES was compared with that of AA alone and the physical mixture, showing a better permeability than the others at the same concentrations. THEDES containing AA in the form of a serum formulation was effective in improving skin condition in female participants. Therefore, the proposed AA and Bet-based DES system might serve as a multifunctional drug delivery system that not only increases the solubility of AA but also improves skin permeability. Furthermore, our method showed potential application with GCL and Bet-based DES, which indicates that it might be extended to develop other versatile drug delivery systems based on THEDES. In conclusion, our findings highlight the potential of THEDES as a promising platform for enhancing API solubility and skin permeability, paving the way for the development of effective topical formulations.

## Figures and Tables

**Table 1:** Description of the components in the THEDES.

Component			Molar Ratio	Abbreviation	Appearance
1	2	3			
Ascorbic acid	Betaine	Water	3:1:8 ^a^	-	Solid paste
			2:1:6 ^b^	AA:Bet:H_2_O (2:1:6)	Clear liquid at room temperature
			1:1:2	AA:Bet:H_2_O (1:1:2)	
			1:5:8	-	Solid paste

^a,b^ The mixtures were prepared by increasing the pH of the solutions; 5 and 3 wt% potassium hydroxide were added to a and b, respectively.

## Data Availability

Data is contained within the article and Supplementary Materials.

## Supplementary Materials

The following are available online at https://www.mdpi.com/article/10.3390/pharmaceutics16050687/s1. Figure S1: HPLC-UV chromatograms of AA and THEDES. Figure S2: ^1^H NMR spectra of AA, Bet, and THEDES. Figure S3: Skin permeability of AA in various THEDES formulations compared to a commercial product. Figure S4: Skin permeability of GCL in the form of THEDES, with comparison to a GCL-only solution, and evaluation of daily exfoliation rate with representative images. Table S1. The age and skin type of subjects in in vivo human study.
