# Supplementary material for: Formulation of Ascorbic Acid and Betaine-based Therapeutic Deep Eutectic System for Enhanced Transdermal Delivery of Ascorbic Acid
Source: Pharmaceutics. 2024 May 20;16(5):687. doi: 10.3390/pharmaceutics16050687 (PMC11124945; doi:10.3390/pharmaceutics16050687)
Supplement: Supplementary file 1 [file pharmaceutics-16-00687-s001.zip › pharmaceutics-2996634-supplementary.pdf]

Supporting Information

Formulation of Ascorbic acid and Betaine-Based Therapeutic Deep Eutectic System for Enhanced Transdermal Delivery of Ascorbic Acid

Ji-Eun Song, Seung-Hyun Jun *, Joo-Yeon Ryoo and Nae-Gyu Kang *

R&D Center, LG Household and Health Care (LG H&H), 70, Magokjungang 10-ro, Gangseo-gu, Seoul 07795, Republic of Korea; sos6934@lghnh.com (J.-E.S.); ryoojy93@lghnh.com (J.-Y.R.)

* Correspondence: junsh@lghnh.com (S.-H.J.); ngkang@lghnh.com (N.-G.K.); Tel.: +82-2-6980-1239 (S.-H.J.); +82-2-6980-1533 (N.-G.K.)

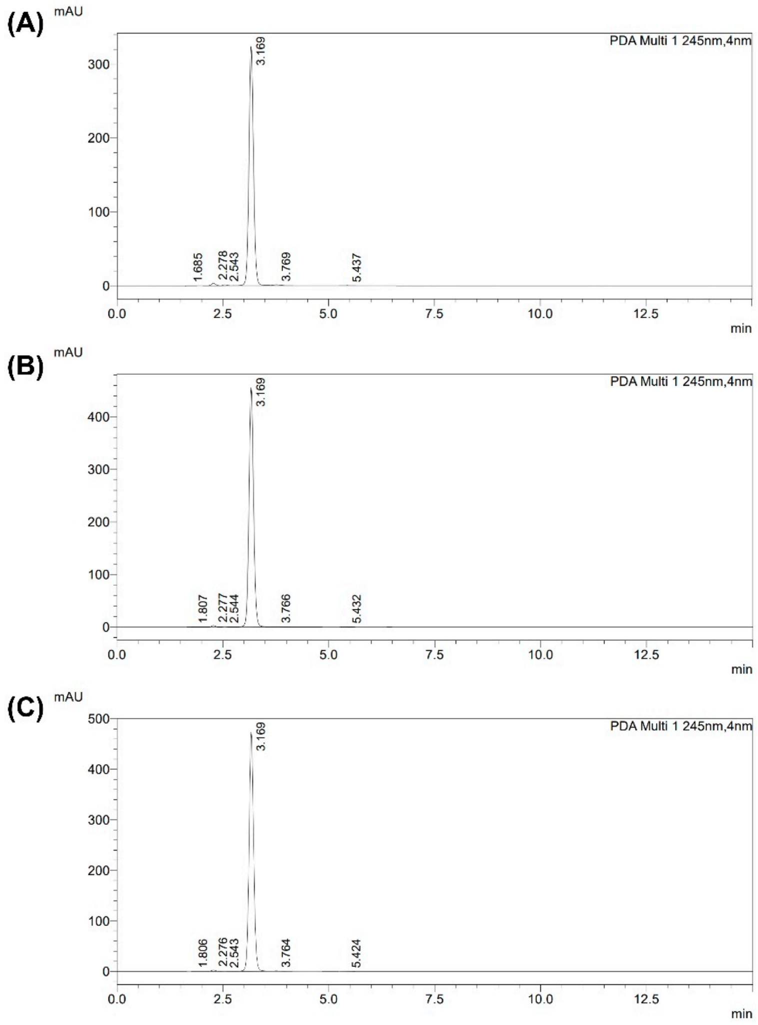

Figure S1. HPLC-UV chromatograms of (**A**) AA, (**B**) the THEDES (AA:Bet:H_2O at a molar ratio of 1:1:2), and (**C**) the THEDES (AA:Bet:H_2O at a molar ratio of 2:1:6). The retention time for standard AA was recorded as 3.169 min, and detection of AA in THEDES at the same retention time was confirmed. UV detection wavelength was 245 nm.

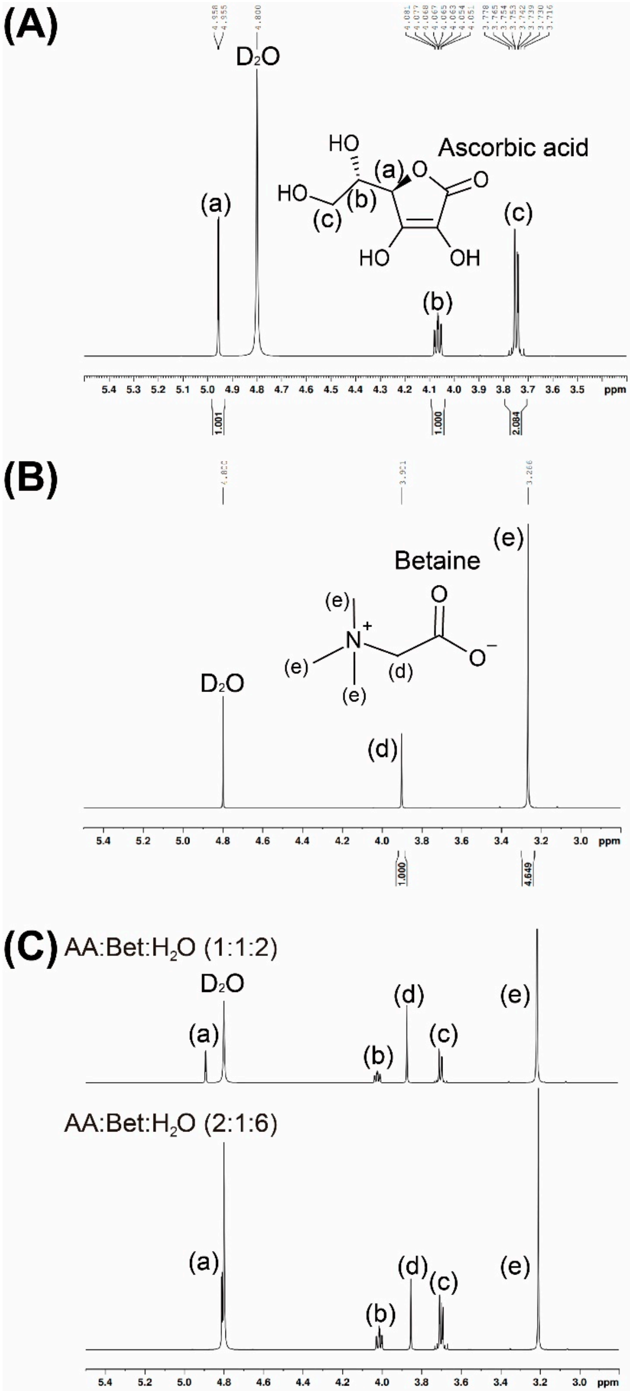

Figure S2. NMR spectra of (A) AA, (B) Bet, and (C) the THEDESs (AA:Bet:D_2O at a molar ratio of 1:1:2 and 2:1:6). Each sample was diluted with D_2O prior to measurement. The ^1H spectra were recorded at 500 MHz on a Bruker AvanceIII 500 spectrometer (Bruker, Ettlingen, Germany). The solvent used was deuterium oxide (D_2O) and 3-(trimethylsilyl)propionic-2,2,3,3-d4 acid sodium salt (TSP) was used as a reference with the chemical shift values are quoted in part per million (ppm).

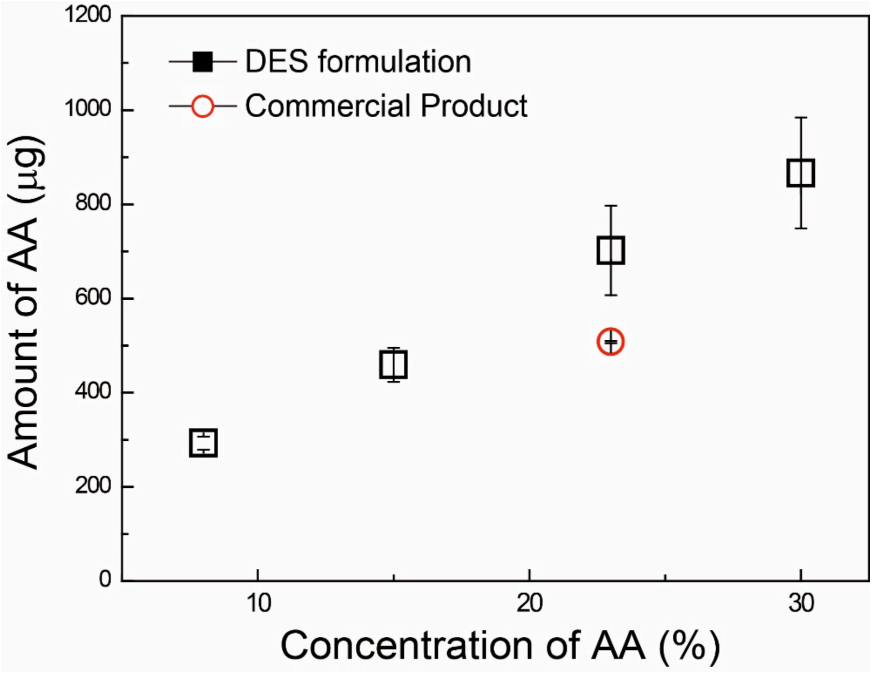

Figure S3. The skin permeability of ascorbic acid (AA) according to the concentrations of AA (8, 15, 23, and 30 wt% of AA) in THEDES (AA:Bet:H_2O at a molar ratio of 2:1:6)-containing serum formulations. Additionally, a water-based vitamin C commercial product was also compared.

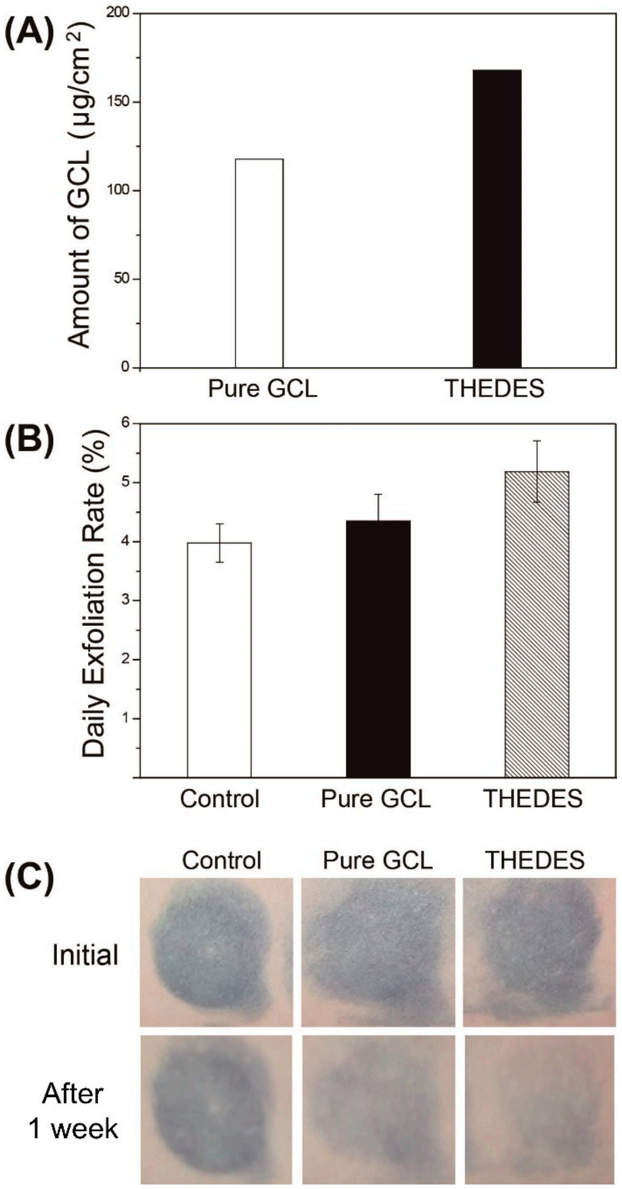

Figure S4. (A) The amount of GCL delivered to the skin after application of the solution containing THEDES (GCL:Bet (3:1), 2 wt% GCL) for 17 hours. For comparison, GCL single-component solution (2 wt%) without Bet (i.e., GCL-alone group) was also compared. **(B)** Daily exfoliation rate and **(C)** representative images according to the evaluation of *in vivo* exfoliation test.

In vivo exfoliation test

This study was approved by the Ethics committee of LG H&H Institutional Review Board (LGHH-20221117-AA-01-01). Prior to participation in the study, human test subjects were informed of possible side effects. Total Seven people who agreed to the study were tested. To evaluate the *in vivo* exfoliation rate, three circles with a diameter of 1.8 cm were stained with henna on the upper arm of each subject. Nothing was applied to one stained circle to compare individual-specific exfoliation rates (Control). Formulations containing THEDES (GCL:Bet (3:1), 2 wt% GCL) and Pure GCL (without Bet, 2 wt% GCL) were applied to the other two stained circles, respectively. Skin brightness in the stained area was measured using a chroma meter(CHROMA METER CR-400, DATA PROCESSOR DP-400). Subjects applied approximately 0.1 g of formula twice a day to the stained circular area. Changes of brightness of stained skin according to the progress of exfoliation were measured by the chroma meter once a

week for 3 weeks. The average daily exfoliation rate was calculated by dividing the difference in the brightness of the stained skin before and after the application of the formulation by the difference in the brightness of the skin before (original skin) and one day after staining.

Table S1. The age and skin type of subjects in *in vivo* human study

Number	Age	Texture of skin
1	26	Mix
2	30	Dry
3	43	Oily
4	35	Dry
5	32	Mix
6	30	Mix
7	28	Dry
8	36	Dry
9	25	Mix
10	30	Mix
